# The Causal Relationships and Therapeutic Targets of Plasma Proteins in Ankylosing Spondylitis

**DOI:** 10.3390/biomedicines13020306

**Published:** 2025-01-27

**Authors:** Pengfei Wen, Mingyi Yang, Yidian Wang, Yuyu Niu, Peng Yang, Shouye Hu, Lin Liu, Zhi Yang

**Affiliations:** 1 Department of Joint Surgery, Honghui Hospital, Xi’an Jiaotong University, Xi’an 710049, China; ymy25808@163.com (M.Y.); honghui3615@163.com (Y.W.); yangpeng_9686@163.com (P.Y.); hushouye2021@126.com (S.H.); liulin183092@163.com (L.L.); 2 Graduate School, Xi’an Medical University, Xi’an 710021, China; 15029216998@163.com

**Keywords:** ankylosing spondylitis, mendelian randomization, plasma proteins, causal relationship, therapeutic targets

## Abstract

**Objective:** The purpose of this study was to assess the causal effects of circulating plasma proteins on ankylosing spondylitis (AS) and to explore potential therapeutic targets. **Methods:** The study used protein quantitative trait loci (pQTLs) for thousands of plasma proteins from nine genome-wide association studies (GWAS) as instrumental variables. The relationship between genetically predicted plasma proteins and AS was assessed through Mendelian randomization (MR) analysis. Further analyses, including colocalization analysis, Steiger filtering analysis, protein-altering variant assessment, protein–protein interaction (PPI), and pathway enrichment analysis, were conducted to validate the robustness and causal direction of the results, as well as to investigate the protein functions and potential drug targets. **Results:** Nine unique proteins were found to have strong causal associations with AS. Steiger filtering analysis confirmed that all associations identified by MR analysis have a direct causal link from the proteins to AS. Colocalization analysis identified four unique proteins—Interleukin-6 receptor alpha (IL-6Rα), Interleukin-23 receptor (IL-23R), Thrombospondin-2 (THBS2), and Interleukin-1 receptor type 2 (IL-1R2)—that share the same causal variants with AS. PPI and pathway enrichment analysis revealed the potential roles of these proteins in inflammatory responses and immune regulation. Moreover, these proteins were valuable drug targets or considered druggable. **Conclusions:** This study has identified multiple plasma proteins associated with AS, revealing the important roles of these proteins in the pathogenesis of AS and providing potential therapeutic targets for AS.

## 1. Introduction

Ankylosing spondylitis (AS) is a chronic inflammatory disease with an incidence and prevalence worldwide ranging from 0.05 to 1.4 per 10,000 person-years and from 0.1% to 1.4%, respectively [1]. The disease usually develops in young individuals, especially between the ages of 16 and 35, and is more common in males than females, with a ratio of approximately 2:1 to 3:1 [2]. The characteristic symptoms of AS include chronic lower back pain and stiffness, which are notably worse in the mornings or after prolonged periods of inactivity [3]. As the condition progresses, patients may experience ossification and fusion of the spine, leading to restricted movement and a significant impact on work and daily life [4]. AS affects not only the axial skeleton but can also involve other areas such as the eyes, heart, lungs, and nervous system, presenting patients with a range of health challenges [5]. Regrettably, the pathogenesis of AS is still not fully understood, which limits the development of preventive, therapeutic, and interventional strategies for the disease. Therefore, further exploration of the pathogenesis of AS and the identification of new therapeutic targets are important for improving patient outcomes and quality of life.

Human circulating plasma proteins are key components of plasma, playing an essential role in maintaining physiological balance, participating in metabolic regulation, and in the diagnosis and treatment of diseases [6]. In-depth research on plasma proteins can help to reveal the complexity of diseases and provide a scientific basis for the development of new therapeutic strategies. Mendelian randomization (MR) is a genetic epidemiological tool that utilizes genetic variations as instrumental variables (IVs) to assess the causal relationship between exposure factors and outcomes [7]. This method is based on Mendelian laws of inheritance, particularly the principle of random segregation of alleles, which ensures that genetic variations are randomly allocated before birth and are independent of lifestyle and environmental factors [7]. Compared to traditional randomized controlled trials and observational studies, it has the advantage of reducing confounding factors and avoiding the impact of reverse causality [8,9]. In recent years, advances in plasma proteomics have yielded information on protein quantitative trait loci (pQTLs) for thousands of circulating proteins. By identifying the association between genetic variations and plasma protein levels through genome-wide association studies (GWAS), researchers can evaluate the potential role of these proteins in disease development, providing new perspectives for studying disease causality, discovering new biomarkers, and identifying therapeutic targets [10,11].

In this study, we used a large-scale, two-sample MR approach to assess the causal effect of circulating plasma proteins on AS. Colocalization analysis and Steiger filtering were conducted to identify robust proteins associated with AS. Subsequently, we carried out protein–protein interaction analysis, pathway enrichment analysis, and drug target assessment to deepen the understanding of AS pathogenesis and to identify potential therapeutic targets.

## 2. Methods

### 2.1. Study Design and Ethics

**Figure 1:**
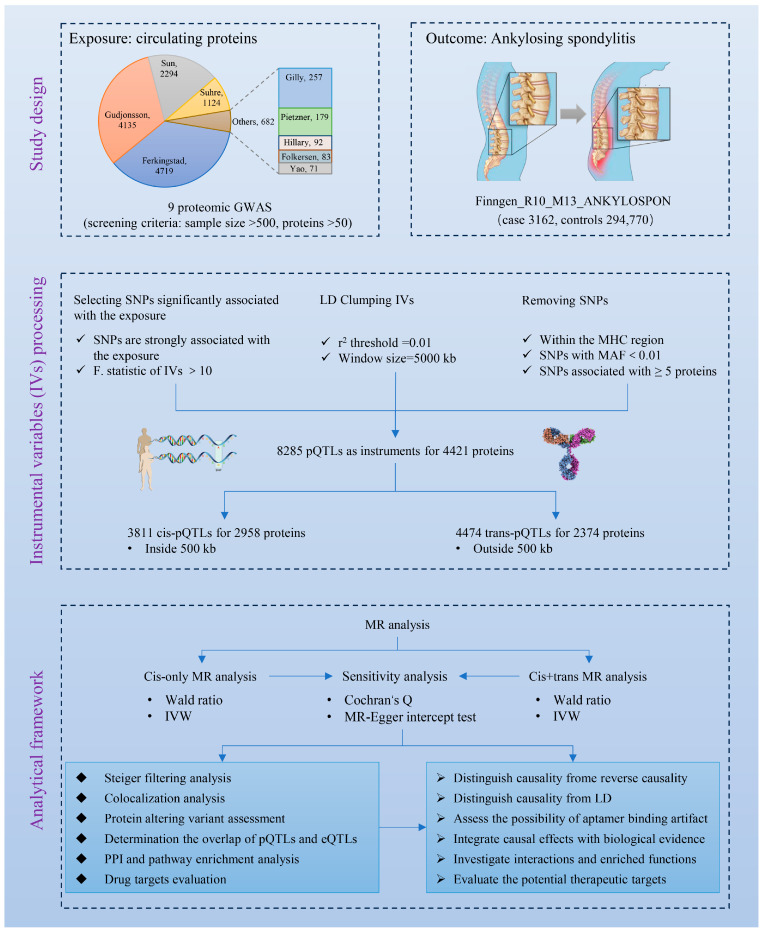
Flowchart of the study design for evaluating the effect of the plasma proteome on ankylosing spondylitis. This flowchart consists of three sections, including study design, instrumental variables processing, and data analysis. GWAS, genome-wide association study; SNP, single nucleotide polymorphisms; LD, linkage disequilibrium; MHC, major histocompatibility complex; MAF, minor allele frequency; pQTLs, protein quantitative trait loci; eQTLs, expression quantitative trait loci; MR, Mendelian randomization; IVW, inverse variance weighting; PPI, protein–protein interaction.

In this study, we focused on analyzing the potential links between genetically predicted circulating plasma proteins and AS. The study design is presented in Figure 1. The data used in the research were derived from public databases and existing publications, which are freely accessible and have been approved by ethical committees.

### 2.2. Exposure and Outcome Data Sources

We extracted summary statistics of plasma protein pQTLs from nine GWAS based on stringent selection criteria (samples exceeding 500 individuals, with more than 50 proteins measured), involving 68,345 individuals [6,12,13,14,15,16,17,18,19]. The specific details of the GWAS data are provided in Supplementary Table S1. These pQTLs were used as candidate IVs after a series of screening processes to ensure they met the three major assumptions of IVs [20]. (i) We selected pQTLs linked to protein using the recommended *p*-value thresholds in the corresponding studies as IVs and applied the F-statistic (beta2/se2) to assess the strength of the IVs, excluding pQTLs with F-values less than 10 to reduce bias. (ii) Due to the complex linkage disequilibrium (LD) patterns in the major histocompatibility complex (MHC) region (chromosome 6: from 26 Mb to 34 Mb), we removed single nucleotide polymorphisms (SNPs) within this region. (iii) Independent SNPs for each protein were identified through LD clumping (with a threshold of r^2^ > 0.01 and distances of less than 5000 kb upstream and downstream). (iv) SNPs associated with five or more proteins were excluded due to their high pleiotropy. (v) SNPs with a minor allele frequency (MAF) less than 0.01 and those that explain the outcome more than the exposure were removed.

The outcome variable of the study is based on the AS GWAS summary statistics from the Finnish R10 dataset, covering 3162 AS cases and 294,770 control individuals of European ancestry. For more details, please refer to Supplementary Table S2 (https://storage.googleapis.com/finngen-public-data-r10/summary_stats/finngen_R10_M13_ANKYLOSPON.gz, accessed on 21 June 2024).

### 2.3. MR Analysis

The genetically predicted plasma proteins were considered as the exposures and AS as the outcome. We categorized these IVs into cis-pQTLs and trans-pQTLs, with cis-pQTLs specifically referring to those located within a 500 kb window of the protein-coding sequence and trans-pQTLs referring to those situated outside a 500 kb window of the protein-coding gene [11]. This classification helped us to more accurately identify genetic variations associated with the expression of specific proteins. In MR analysis, we utilized both cis and all (cis + trans) pQTLs as IVs. When only one pQTL was available, the MR effect was estimated using the Wald ratio; when multiple pQTLs were present, the relationship was assessed using the inverse variance weighting (IVW) approach [11]. Additionally, to address the issue of pleiotropy in IVs, we examined the protein–trait associations supported by MR evidence, excluding those IVs associated with more than one protein. Furthermore, sensitivity analyses were performed to ensure the robustness of the results. If heterogeneity among multiple instruments was detected in Cochran’s Q test, the IVW random effects model was applied as the outcome [21]. Upon detecting horizontal pleiotropy using the MR-Egger method, the analysis proceeded with this method because it could correct the effects of instruments’ pleiotropy [22].

The entire MR analysis was conducted in the R v4.2.3 environment, using the “TwoSampleMR” R package. We employed the false discovery rate (FDR) for multiple testing corrections and set the significance threshold at P_fdr_ < 0.05. Forest plots visually conveyed the magnitude of the effect and statistical significance.

### 2.4. Steiger Filtering Analysis

Steiger filtering analysis is a method used in MR studies to assess the robustness of results, particularly to detect whether observed associations may be distorted due to reverse causality [11]. Steiger filtering was performed using the “TwoSampleMR” R package for MR associations that passed the FDR threshold. The results are presented as categorical variables, where “True” indicates that the effect direction from exposure to outcome is consistent at the *p* < 0.05 level; “False” indicates that the effect direction is opposite at the *p* < 0.05 level, which may suggest reverse causality; “Uncertain” indicates that the effect direction cannot be determined at *p* ≥ 0.05, and the robustness of the results is questionable [23].

### 2.5. Colocalization Analysis

Bayesian colocalization analysis is a statistical method used to detect whether two traits share the same causal variant in the genome. This method is crucial for addressing the potential false-positive results caused by LD in MR analysis [24]. To identify plasma proteins with potential causal relationships with AS as much as possible, all data with *p*-values less than 0.05 from MR results in the colocalization analysis were used. Colocalization analysis utilizing the R package “coloc” allowed us to assess the posterior probability that a single variant in a genomic locus affects both traits. Five different hypotheses were considered for colocalization analysis, where hypothesis 4 suggests that the two traits are associated with the same genetic variation within a region. If the posterior probability exceeds 0.75, it is considered strong evidence of colocalization, thereby strengthening the causal relationship between the two traits [20]. However, if the posterior probability of hypothesis 3 is close to 1, it indicates that the MR results are confounded by LD and need to be excluded from the study [24].

### 2.6. Annotation of Protein-Altering Variations in Cis-pQTLs

Protein-altering variations (PAVs) are genetic variations that affect the protein-coding genes and their surrounding regions. In proteomics research, affinity-based techniques may be disrupted by PAVs because they may alter the binding of proteins to their ligands rather than the expression levels of proteins, leading to misinterpretation of cis-pQTLs [6,14]. To avoid this artifact, the Ensembl Variant Effect Predictor (https://github.com/Ensembl/ensembl-vep, accessed on 25 June 2024) was used to annotate cis-pQTLs with MR evidence to identify possible PAVs. A variant is considered a PAV if it is predicted to potentially affect the protein structure or function, such as coding sequence variants, frameshift variants, missense variants, etc., or is directly predicted to be a PAV. Furthermore, if a variant is in LD (r^2^ ≥ 0.8) with a known PAV, it may also be considered a PAV [6].

### 2.7. Overlap Assessment of pQTLs and Expression Quantitative Trait Loci (eQTLs)

The overlap assessment between pQTLs and eQTLs is a method to study the genetic link between gene expression and protein levels. By analyzing the overlap between genetic variations that affect plasma protein levels and those that affect gene expression, the correlation between the two can be revealed [11]. Using data from the Genotype-Tissue Expression (GTEx) portal (Version 8, https://www.gtexportal.org, accessed on 26 June 2024), we extracted eQTLs and assessed whether the pQTLs with MR evidence and their proxy variants (r^2^ ≥ 0.8) had corresponding significant eQTLs, determining whether the allelic effect directions were consistent. Furthermore, through the Open Targets Genetics platform (https://genetics.opentargets.org, accessed on 26 June 2024), we further evaluated the overlap between pQTLs and AS-associated eQTLs to explore how pQTLs influence protein expression at the transcriptional level and to enhance the biological interpretability of pQTLs.

### 2.8. Protein–Protein Interaction and Functional Enrichment Analysis

To deeply explore the role and function of MR-prioritized proteins in the pathogenesis of AS, we employed a series of bioinformatics tools and databases. Initially, a protein–protein interaction (PPI) network was constructed through the STRING database (V11.5, https://string-db.org, accessed on 27 June 2024), which covers 9.6 million proteins and 13.8 million PPI records across 2031 species, providing powerful support for analyzing potential connections between proteins [25]. Subsequently, using the ’ClusterProfiler’ R package (V 4.1.0), Gene Ontology (GO) and Kyoto Encyclopedia of Genes and Genomes (KEGG) pathway analysis were performed to identify the biological functions and pathways associated with these proteins [25]. The GO analysis revealed the information related to the genes encoding proteins in terms of cellular component, biological process, and molecular function, while the KEGG analysis clarified the specific biological pathways and their functions involving clusters of genes.

### 2.9. Mapping MR-Prioritized Proteins to Drug Targets

Human proteins as key targets in drug development play a crucial role in the discovery of new medicines. To assess whether MR-prioritized proteins overlap with the druggable genome, we mapped these proteins to the list of 4479 druggable genes proposed by Finan et al. [26]. These drug targets are categorized into three tiers: Tier 1 includes targets of approved drugs and clinical-stage candidates; Tier 2 comprises target genes encoding known bioactive drug-like small molecule binding partners, as well as target genes with ≥50% identity to approved drug targets; Tier 3 consists of genes encoding secreted or extracellular proteins with some similarity to approved drug targets, further subdivided based on their proximity to GWAS SNPs and extracellular location. In our analysis, the focus was on the priority levels of MR-prioritized proteins within the druggable genome and whether they are targeted by small molecules or biotherapeutics.

In addition, we annotated MR-prioritized proteins for drug target potential using the Therapeutic Target Database (accessed via http://db.idrblab.net/ttd, accessed on 27 June 2024), which lists 3578 drug targets, including successful targets, clinical trial targets, preclinical/patented targets, and research targets.

## 3. Results

### 3.1. Selection of Genetic Instruments

Figure 1 illustrates the process by which genetic instruments were selected. From nine proteomic GWAS, pQTLs were filtered to construct these instruments. A total of 8285 pQTLs were retained, linking to 4421 proteins, including 2518 unique proteins, as instruments for MR analysis (Supplementary Table S3). These genetic instruments were categorized into cis-pQTLs and trans-pQTLs, with 3811 cis-pQTLs associated with 2958 proteins (1558 unique proteins) and 4474 trans-pQTLs associated with 2374 proteins (1763 unique proteins). Among the 4421 proteins with valid genetic instruments, 2047 were affected only by cis-pQTLs, 1463 only by trans-pQTLs, and 911 by both cis- and trans-pQTLs.

### 3.2. Causal Effects of Plasma Proteins on AS

**Figure 2:**
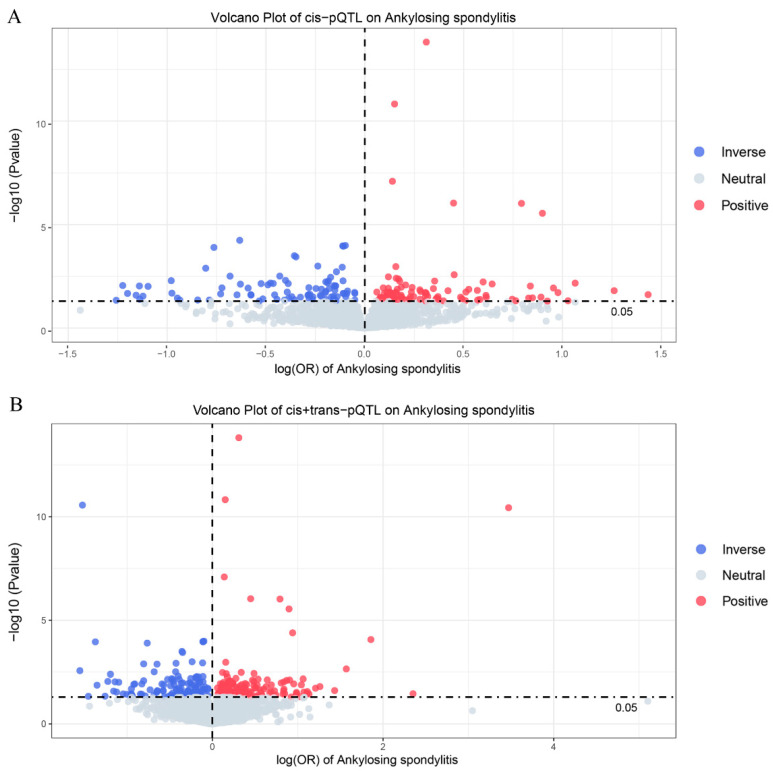
Volcano plot of the association between plasma proteins and ankylosing spondylitis (AS). (**A**) Only cis-pQTL MR analysis. (**B**) Combined cis + trans-pQTL MR analysis. Each point in the volcano plot represents a specific protein. The color of the points reflects the correlation with the risk of AS. Red points indicate a positive correlation, blue points suggest a negative correlation, and gray points indicate no significant correlation.

**Figure 3:**
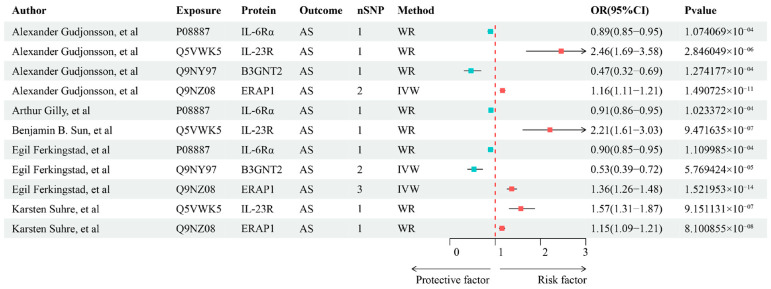
Protein–phenotype associations identified by Mendelian randomization using cis-pQTLs. IL-6Rα, Interleukin-6 receptor subunit alpha; IL-23R, Interleukin-23 receptor; B3GNT2, beta-1,3-N-acetylglucosaminyltransferase 2; ERAP1, endoplasmic reticulum aminopeptidase 1; AS, ankylosing spondylitis; pQTLs, protein quantitative trait loci; WR, Wald ratio; IVW, inverse variance-weighted; FDR, false discovery rate; CI: confidence interval; OR: odds ratio [6,12,13,18].

In this study, the focus was initially on cis-pQTLs, as they are more likely to influence specific biological effects. We used these cis-pQTLs to systematically assess the potential causal relationship between plasma proteins and AS. Cis-MR analysis indicated that 188 proteins (141 unique proteins) were significantly associated with AS (*p*-value < 0.05), suggesting that these proteins may be closely related to the pathogenesis of AS (Figure 2A). Following FDR threshold correction, 11 proteins (four unique proteins) were further confirmed to have a strong association with AS (Figure 3 and Supplementary Table S4). Sensitivity cis-pQTLs MR analysis showed consistency with the main analysis results, with no new significant protein–trait associations emerging or disappearing (Supplementary Table S5).

**Figure 4:**
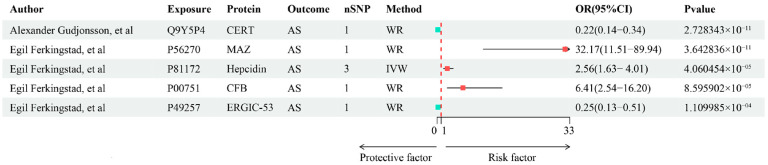
Protein–phenotype associations identified by Mendelian randomization using trans-pQTLs. CERT, Ceramide transfer protein; MAZ, Myc-associated zinc finger protein; CFB, Complement factor B; ERGIC-53, endoplasmic reticulum–Golgi intermediate compartment protein 3; AS, ankylosing spondylitis; pQTLs, protein quantitative trait loci; WR, Wald ratio; IVW, inverse variance-weighted; FDR, false discovery rate; CI: confidence interval; OR: odds ratio [12,13].

To improve the reliability of the associations between proteins and phenotypes, trans-pQTLs were further incorporated into the MR analysis. In the analysis using all (cis + trans) pQTLs as instruments, a total of 289 proteins (239 unique proteins) were observed to have significant associations with AS (*p*-value < 0.05) (Figure 2B and Supplementary Table S6). After FDR correction, 16 proteins (nine unique proteins) were identified with significant associations with AS, including 5 proteins (five unique proteins) discovered through trans-MR analysis (Figure 4). In sensitivity analysis, all significant associations detected in the main analysis remained stable, with no emergence or disappearance of new significant protein–trait associations (Supplementary Table S7).

Through Steiger filter analysis, it was confirmed that all associations identified through MR analysis support the causal direction from proteins to AS (Supplementary Table S8). This indicates that these associations are more likely caused by changes in proteins rather than AS causing changes in proteins.

### 3.3. Colocalization of pQTLs with AS Risk Loci

To eliminate the potential confounding effects of LD on the MR results, a colocalization analysis was conducted. This analysis aimed to confirm whether the associations between proteins and AS were caused by the same causal variant. In the cis-MR prioritized associations, eight proteins (four unique proteins) demonstrated significant colocalization evidence (Supplementary Table S9 and Figure S1). Additionally, in the trans-MR prioritized associations, no significant colocalization evidence was observed (Supplementary Table S10). This indicates that in these instances, the associations may not be caused by the same causal variant.

### 3.4. PAV Assessment for Cis-pQTLs and Overlap Between pQTLs and eQTLs

We conducted a PAV assessment for pQTLs that showed evidence in colocalization analysis. Among the four predicted cis-pQTL-related unique proteins, pQTLs in two unique proteins were either PAVs themselves or in strong LD (r^2^ > 0.8), with PAVs affecting the corresponding genes (Supplementary Table S11). Eight previously identified cis-pQTLs with MR evidence were employed as IVs. Based on the GTEx project data, no significant overlap was found with eQTLs acting in the same direction as pQTLs or their proxies. However, when we retrieved eQTLs in AS-associated tissues or cells from the Open Targets Genetics database, three cis-pQTLs overlapped with eQTLs related to monocytes were discovered (Supplementary Table S12).

### 3.5. Investigation of PPI and Enrichment Pathways of MR-Prioritized Proteins

To further investigate the interactions among MR-prioritized proteins and their roles in the pathogenesis of AS, PPI analysis and pathway analysis were conducted. Although a medium confidence interaction score threshold of 0.4 was applied to capture interactions, the results indicated that the four selected proteins did not form a distinct interaction network (Supplementary Figure S2). GO enrichment analysis revealed that these proteins may be related to the positive regulation of Janus kinase activity. Furthermore, KEGG pathway analysis showed that these proteins were associated with cytokine–cytokine receptor interactions (Supplementary Table S13).

### 3.6. Assessment of Drug Targets for MR-Prioritized Proteins

Recognizing the significance of human proteins as therapeutic targets in drug development, an evaluation was conducted on MR-prioritized proteins to determine their potential as drug targets. Firstly, these proteins were compared with the druggable gene list established by Finan and colleagues [26]. The results revealed that three of the four proteins assessed have been identified as drug targets, with one classified at Tier 1 level and the other two at Tier 3B level (Table 1). Further investigation using the Therapeutic Target Database indicated that three proteins are targets of existing or potential drugs [11]. Specifically, two of the proteins were successful targets while the other was a clinical trial target. Additionally, it was noted that two-thirds of the proteins with targeted drugs in the Therapeutic Target Database overlap with the druggable genes identified by Finan and colleagues (Table 1 and Supplementary Table S14).

## 4. Discussion

In this study, the potential causal associations between thousands of plasma proteins and AS were explored in depth by the MR method using data from nine large-scale proteomic GWAS. The findings revealed significant associations with 239 unique proteins, with 9 unique proteins demonstrating the strongest causal associations after FDR adjustment. Further colocalization analysis identified four unique proteins in the cis-MR results: Interleukin-6 receptor subunit alpha (IL-6Rα), Interleukin-23 receptor (IL-23R), Thrombospondin-2 (THBS2), and Interleukin-1 receptor-like 2 (IL-1R2). Subsequently, we performed a series of critical analyses to verify the robustness and causal direction of the results and to explore the potential regulatory mechanisms and drug targets of these proteins. These proteins were involved in key biological functions and pathways, such as inflammatory response, immune regulation, and cell signaling, thereby providing a foundation for the study of AS pathogenesis and the development of future therapeutic strategies.

**Figure 5:**
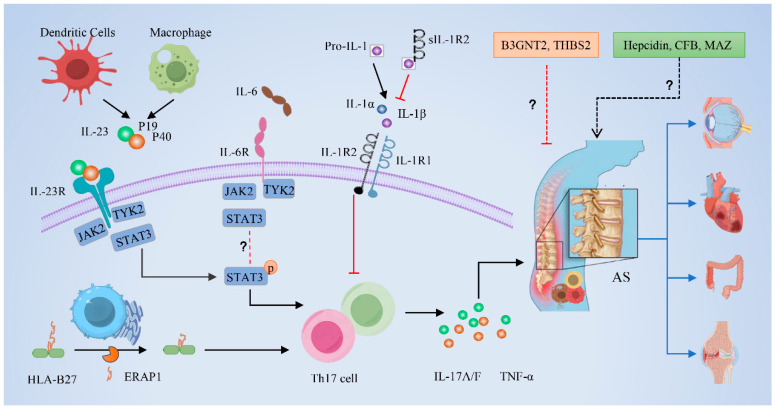
Cytokines and molecular pathways associated with ankylosing spondylitis (AS). Black arrows indicate a positive regulatory effect, suggesting that these molecules or factors promote the development of AS; red lines denote a negative regulatory role, implying that these molecules or factors may inhibit the progression of AS. Dashed lines and question marks in the figure represent molecules or mechanisms that are not yet fully understood or require further investigation. AS, ankylosing spondylitis; B3GNT2, beta-1,3-N-acetylglucosaminyltransferase 2; THBS2, Thrombospondin-2; CFB, complement factor B; MAZ, Myc-associated zinc finger protein; sIL-1R2, soluble Interleukin-1 receptor 2; Pro-IL-1, Pro-interleukin-1; IL-6, Interleukin-6; IL-1α, Interleukin-1 alpha; IL-1β, Interleukin-1 beta; IL-23, Interleukin-23; IL-6R, Interleukin-6 receptor; IL-1R2, Interleukin-1 receptor 2; IL-1R1, Interleukin-1 receptor 1; TYK2, Tyrosine kinase 2; JAK2, Janus kinase 2; STAT3, signal transducer and activator of transcription 3; HLA-B27, human leukocyte antigen B27; ERAP1, endoplasmic reticulum aminopeptidase 1; IL-17A/F, Interleukin-17A/F; TNF-α, tumor necrosis factor alpha.

In our cis-MR analysis results, IL-23R and endoplasmic reticulum aminopeptidase 1 (ERAP1) were positively correlated with AS. IL-23, which is significantly elevated in the peripheral blood and bone tissue of AS patients, can bind to IL-23R and participate in the regulation of the immune system [27]. The activation of IL-23R can drive the production of IL-17 by Th17 cells. The IL-23/IL-17 axis-mediated immune response can exacerbate inflammatory responses in the pathogenesis of AS [28,29] (Figure 5). A meta-analysis that included 43 studies with 13,917 cases and 19,849 controls found that genetic susceptibility to AS is associated with nine SNPs of IL-23R in the general population [30]. The susceptible alleles of IL-23R are also related to psoriatic arthritis (PsA) and inflammatory bowel disease (IBD) [28]. Overexpression of IL-23 cytokines in the liver of mice can lead to the rapid onset of peripheral inflammation, which then spreads to the synovium and bones, causing the destruction of multiple joints [31]. This indicates that the level of IL-23 is related to the activity and severity of the disease. The recent published literature has shown that IL-23 p40 blockers and p19 inhibitors are effective against peripheral PsA, peripheral synovitis, and systemic inflammation [32,33]. In addition, it has been found that PDE4 blockers can reduce IL-23 secretion, thereby lowering IL-17 to control inflammation [34].

ERAP1 is an aminopeptidase involved in the processing of peptides presented by major histocompatibility complex (MHC) class I molecules in the endoplasmic reticulum [35]. The polymorphisms of ERAP1 are closely associated with the severity and progression of AS [36] (Figure 5). These genetic variations may increase the expression and enzymatic activity of ERAP1, potentially increasing the opportunity for pathogenic peptides to bind with human leukocyte antigen B27 (HLA-B27) and become immunodominant by removing peptides that physiologically bind to HLA-B27 [37]. In AS, ERAP1 is primarily associated with HLA-B27-positive cases, and ERAP1 variants (K528R and Q730E) may alter the peptidome presented by HLA-B27, which is crucial for the disease susceptibility [37,38]. Natural killer (NK) cells play an important role in the development of AS, and ERAP1 has been identified as an AS risk locus that affects the genetic regulation of NK cells [39]. In addition, the single nucleotide polymorphisms of ERAP1 have also been verified in East Asian populations [40]. Thus, ERAP1 may be a potential therapeutic target for AS, and ERAP1 inhibitors or modulators may aid in controlling the inflammatory processes of AS. Chen et al. showed that ERAP1 silencing or inhibition can reduce the surface expression of HLA-B27 free heavy chains. This reduction decreased the activation of the key immune receptor killer cell immunoglobulin-like receptor 3DL2 (KIR3DL2), thereby reducing the production of IL-2 and inhibiting the expansion of AS CD4+ T cells into Th17 cells and the secretion of IL-17A [41]. Our results also demonstrate that ERAP1 protein is positively correlated with the development of AS.

This study showed that Interleukin-6 receptor subunit alpha (IL-6Rα) and beta-1,3-N-acetylglucosaminyltransferase 2 (B3GNT2) were negatively correlated with AS. IL-6Rα is an essential component in the IL-6 signaling pathway, which plays a central role in various inflammatory diseases [42]. Upon binding with its receptor IL-6R, IL-6 can activate the Janus kinase (JAK) and signal transducer and activator of transcription (STAT) signaling pathways, thereby influencing immune cell activation, proliferation, and differentiation [43] (Figure 5). A meta-analysis of 13 case-control studies involving 514 AS patients and 358 controls demonstrated that IL-6 expression levels were significantly elevated in the serum and synovial fluid of AS [44]. Moreover, IL-6 serum levels are closely related to erythrocyte sedimentation rate and C-reactive protein, which can be used to assess the severity and progression of AS [45]. This provides a theoretical basis for the development of therapeutic strategies targeting IL-6Rα. For instance, Tocilizumab and Sarilumab, biological agents targeting IL-6Rα, have shown efficacy in moderate to severe rheumatoid arthritis (RA) [46]. Additionally, Chen et al. concluded that the use of IL6R inhibitors could reduce the risk of AS by drug MR analysis [47]. However, our results differ from these previous studies, suggesting a complex mechanism of IL-6Rα in AS. This negative correlation may indicate that the expression or function of IL-6Rα in AS is regulated or inhibited by other factors. A phase II clinical study indicated that the blockade of IL-6Rα by Sarilumab did not yield significantly effective results in the treatment of AS [48]. Therefore, future research is needed to further explore the exact relationship between IL-6Rα and AS. B3GNT2 is an enzyme involved in the biosynthesis of glycoproteins in humans, primarily involved in the extension and modification of sugar chains, and participates in cell adhesion, signal transduction, and immune responses [49]. GWAS have shown that SNPs reducing the expression of B3GNT2 are associated with autoimmune diseases, including RA and AS [49]. Wang et al. demonstrated that B3GNT2 may affect the development and severity of AS through interaction with HLA-B27 [50]. The specific regulatory mechanisms of B3GNT2 in AS warrant further investigation.

In our trans-MR results, hepcidin, complement factor B (CFB), and Myc-associated zinc finger protein (MAZ) were found to be positively correlated with AS, while ceramide transfer protein (CERT) and endoplasmic reticulum–Golgi intermediate compartment protein 3 (ERGIC-3) were negatively correlated. The roles and mechanisms of these prioritized proteins in AS deserve further exploration. For instance, studies have shown that hepcidin levels were elevated in AS [51]. Hepcidin, as a key regulator of iron metabolism, may be involved in immune responses and inflammatory processes in AS by modulating the iron metabolism and activity of immune cells. CFB is a part of the complement system and is involved in immune response and inflammation by activating the complement cascade. Studies have shown that CFB plays a crucial role in Crohn’s disease [52], RA [53], and lupus nephritis [54], suggesting that CFB may drive the inflammatory process in AS. On the other hand, the negative correlation of CERT and ERGIC-3 with AS may suggest their roles in inhibiting inflammation or regulating immune cell functions.

A series of subsequent analyses were conducted on proteins associated with AS. Bayesian colocalization analysis was employed to explore whether the proteins initially identified might be driven by LD between pQTLs and AS-associated SNPs. The results revealed that only four associations exhibited strong evidence of colocalization, including IL-6Rα, IL-23R, THBS2, and IL-1R2. This finding may be limited by the basic assumption of colocalization analysis, which posits that there is only a single association signal in each region. This assumption may not always hold true in practice, potentially leading to an underestimation of colocalization [55]. The relationships between IL-23R, as well as IL-6Rα, and AS have been extensively discussed in the previous text. Next, we will discuss THBS2 and IL-1R2, which were negatively associated with AS in the study.

THBS2 is a multifunctional extracellular matrix protein that has been found to play a protective role in inflammatory responses [56,57]. The negative correlation of THBS2 with AS suggests that it may act as an inhibitor in the progression of AS by suppressing the release of inflammatory mediators. IL-1R2, an essential component of the IL-1 signaling pathway, is often regarded as a decoy receptor and may be a natural anti-inflammatory factor [58] (Figure 5). In autoimmune diseases, IL-1R2 can control IL-1-driven inflammatory responses and serves as an important internal brake in the later stages of the immune response, limiting IL-1-dependent B cell activation and germinal center reactions, as well as antibody production [58,59]. During the polarization process of Th17 cells, plasmids transfected with IL-1R2 can inhibit the expression of IL-6, transforming growth factor-beta (TGF-β), and ROR-γt, which may subsequently reduce the secretion of IL-17 [60]. Notably, in HLA-B27-negative patients with AS, IL-1R2 showed a protective effect, indicating that it may play a protective role in the pathophysiological mechanisms of AS [61]. This is also supported by our findings. Although the specific mechanism of IL-1R2 in AS requires further research, its potential protective role offers a new strategy for AS treatment.

To rule out the possibility of reverse causality, Steiger filtering analysis was employed, and the results indicated that all associations identified by MR analysis have a direct causal link from proteins to AS. Additionally, the potential impact of PAVs on protein structure and ligand affinity was considered. It was found that the pQTLs for IL-23R and IL-6Rα were PAVs or in LD with PAVs, suggesting caution is needed when interpreting the causal relationship between these proteins and phenotypes [13]. Furthermore, the overlap between pQTLs and eQTLs was assessed to explore whether the association of genetic variants with proteins is mediated by the mRNA expression levels of the corresponding genes. We found that the cis-pQTLs for IL6Rα overlapped with the corresponding eQTLs, while no overlap was observed between trans-pQTLs and their corresponding eQTLs. This suggests that cis-pQTLs have greater biological interpretability, whereas trans-pQTLs may involve more complex regulatory mechanisms. In our study, the MR-prioritized proteins did not form the expected interactive network, which may indicate that their biological roles are independent or mediated by other unidentified proteins. It is encouraging that pathway enrichment analysis linked these proteins to pathways involved in inflammatory responses, providing additional explanation for their biological roles in AS. Finally, a drug target assessment was conducted on proteins with MR evidence, hoping to develop new therapeutic approaches for AS or to provide a basis for the repurposing of existing drugs.

While this study has provided important clues to therapeutic targets for AS, there are some limitations. Firstly, MR analysis relies on existing GWAS data, which may have selection bias in the sample population, potentially limiting the generalizability of the results. Secondly, the study focuses on circulating proteins, whose levels may differ from the protein abundance within tissues or cells, thus restricting our understanding of the impact of protein abundance on AS. Thirdly, Bayesian colocalization analysis typically assumes that there is only one causal variant in a given region or that the main causal variant has a significant effect on the results. If there are multiple causal variants with smaller effects in a region, these variants may not be detected by this method, potentially underestimating the role of certain proteins. Lastly, although associations between proteins and AS have been identified, the specific biological mechanisms behind these associations need to be elucidated by further experimental studies.

## 5. Conclusions

This research has identified nine plasma proteins with significant causal relationships to AS, offering new insights into the pathogenesis of this disease. Notably, IL-6Rα, IL-23R, THBS2, and IL-1R2 play crucial roles in the inflammation and immune response of AS, providing a foundation for advancing AS research and clinical practice. Looking ahead, these novel biomarkers may enhance the ability to diagnose early and monitor disease progression, enabling more timely interventions. Furthermore, developing combination therapies targeting multiple molecular pathways could improve treatment efficacy and reduce side effects. Although gene therapy is still in the experimental stage, it holds therapeutic potential for directly targeting the genetic and molecular mechanisms that cause AS. Additionally, understanding the interaction between genetic susceptibility and environmental factors, such as the gut microbiome, could lead to more personalized treatment strategies. Overall, we believe these efforts will significantly improve the management and treatment of AS, enhancing the quality of life for affected individuals.

## Figures and Tables

**Table 1:** List of the MR-prioritized proteins for drug development.

Uniprot	Protein Names	Gene	Druggability_tier *	Target Type ※	Drugs ※
P08887	IL-6RA	*IL6R*	Tier 1	Successful Target	Sarilumab/Tocilizumab
P35442	Thrombospondin-2	*THBS2*	Tier 3B	/	/
Q5VWK5	IL-23R	*IL23R*	/	Clinical trial Target	Anti-IL-23
Q9HB29	IL-RL2	*IL1RL2*	Tier 3B	Successful Target	Spesolimab

* Based on the druggable genes from Finan et al. ※ Based on the therapeutic target database. IL-6RA, Interleukin-6 receptor subunit alpha; THBS2, Thrombospondin-2; IL-23R, Interleukin-23 receptor; IL-RL2, Interleukin-1 receptor-like 2; MR, Mendelian randomization.

## Data Availability

All data generated or analyzed during this study are included in the article and its Supplementary Information files. Further inquiries can be directed to the corresponding authors.

## Supplementary Materials

The following supporting information can be downloaded at: https://www.mdpi.com/article/10.3390/biomedicines13020306/s1, Figure S1: Colocalization of the pQTLs with AS risk loci. Figure S2: Protein-protein interaction network of the MR-prioritized proteins; Table S1: Source information of the instrumental variables. Table S2: Source information of outcome. Table S3: Information of the instrumental variables. Table S4: Main analyses results of the cis-only MR. Table S5: Sensitivity analyses results of cis-only MR. Table S6: Main analyses results of the cis + trans MR. Table S7: Sensitivity analyses results of of cis + trans MR. Table S8: Steiger filtering analysis for associations identified by main analyses of cis + trans MR. Table S9: Colocalization of the pQTLs that prioritized by the cis-only MR analyses with AS risk loci. Table S10: Colocalization of the pQTLs that prioritized by the trans-only MR analyses with AS risk loci. Table S11: PAV assessment for the cis-pQTLs with MR evidence. Table S12: Overlaping of pQTLs that prioritized by the main analyses of cis + trans MR with eQTLs. Table S13: GO and KEGG pathway enrichment analysis for MR prioritized proteins. Table S14: Drug targets of the MR-prioritized proteins.
