# Supplementary figures and images for: The Causal Relationships and Therapeutic Targets of Plasma Proteins in Ankylosing Spondylitis
Source: Biomedicines. 2025 Jan 27;13(2):306. doi: 10.3390/biomedicines13020306 (PMC11853591; doi:10.3390/biomedicines13020306)

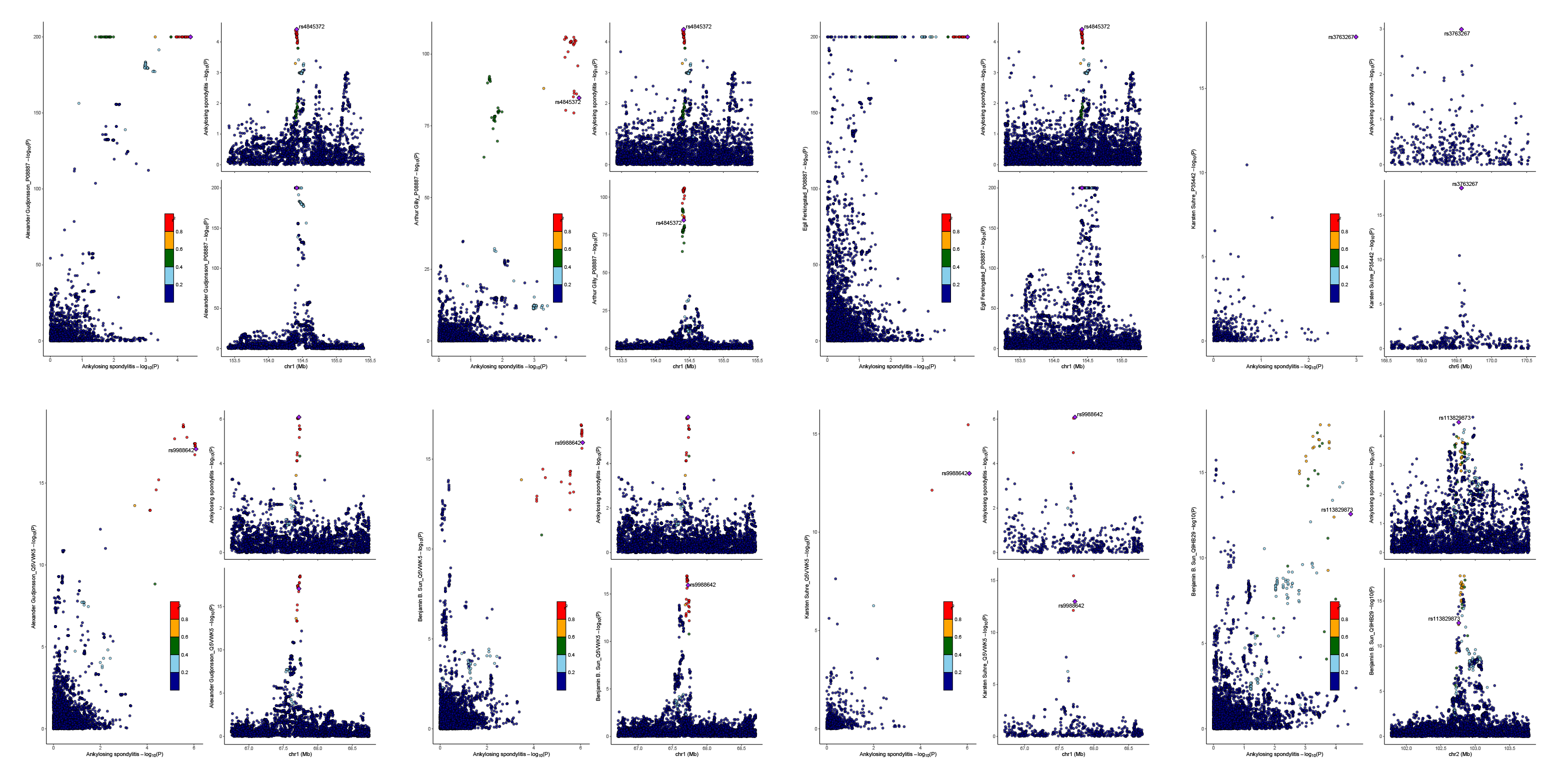

Supplement: Supplementary file 1 [file biomedicines-13-00306-s001.zip › Supplementary Figure S1.tif]

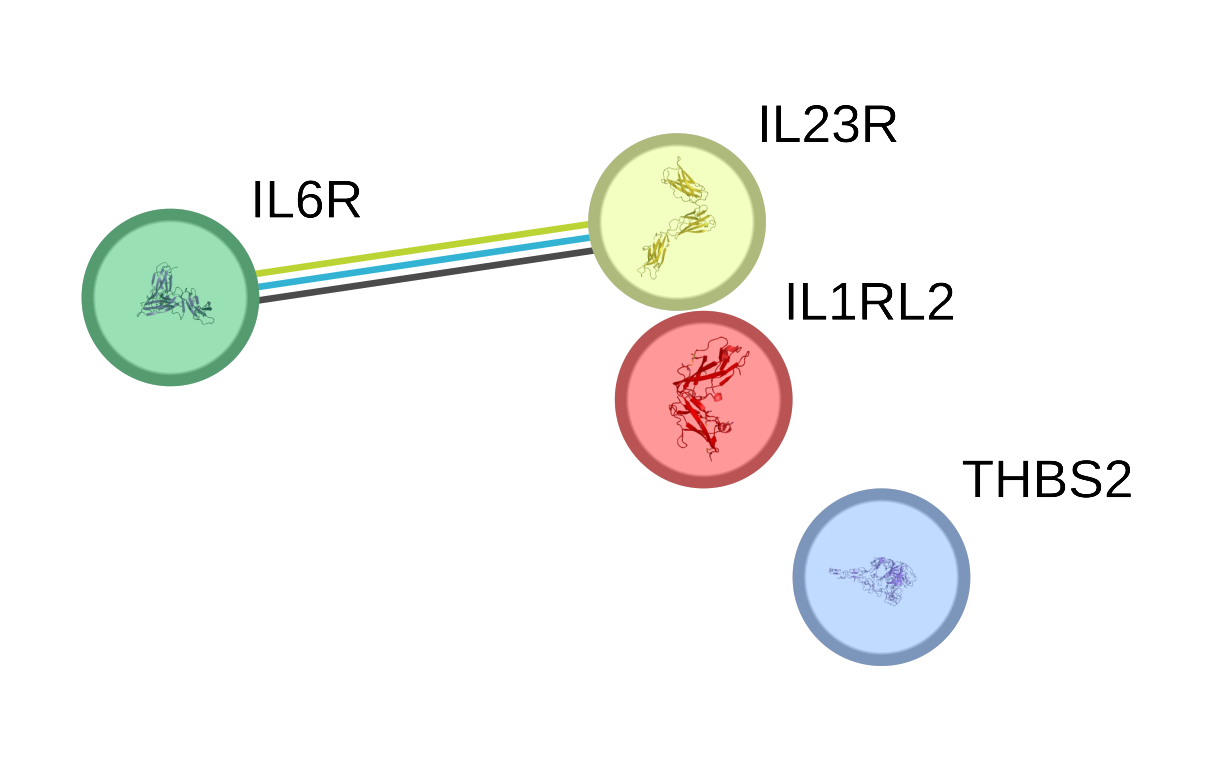

Supplement: Supplementary file 1 [file biomedicines-13-00306-s001.zip › Supplementary Figure S2.tif]
